# Life under stay-at-home orders: a panel study of change in social interaction and emotional wellbeing among older Americans during COVID-19 pandemic

**DOI:** 10.1186/s12889-022-14103-x

**Published:** 2022-09-20

**Authors:** Jielu Lin, Melissa Zajdel, Krystyna R. Keller, Fiona O. Gilpin Macfoy, Philip Shaw, Brenda Curtis, Lyle Ungar, Laura Koehly

**Affiliations:** 1 Social Network Methods Section, Social and Behavioral Research Branch, National Human Genome Research Institute, 31 Center Drive, Building 31, Room B1B37, Bethesda, MD 20892 USA; 2 Neurobehavioral Clinical Research Section, Social and Behavioral Research Branch, National Human Genome Research Institute, Bethesda, MD USA; 3 Technology and Translational Research Unit, Translational Addiction Medicine Branch, National Institute On Drug Abuse, Baltimore, MD USA; 4 Department of Computer and Information Science, University of Pennsylvania, Philadelphia, PA USA

**Keywords:** Social Relationships, Social Support, Stress, COVID-19, Older Adults

## Abstract

**Background:**

Recent research has shown the mental health consequence of social distancing during the COVID-19 pandemic, but longitudinal data are relatively scarce. It is unclear whether the pattern of isolation and elevated stress seen at the beginning of the pandemic persists over time. This study evaluates change in social interaction over six months and its impact on emotional wellbeing among older adults.

**Methods:**

We drew data from a panel study with six repeated assessments of social interaction and emotional wellbeing conducted monthly May through October 2020. The sample included a total of 380 White, Black and Hispanic participants aged 50 and over, of whom 33% had low income, who residing in fourteen U.S. states with active stay-at-home orders in May 2020. The analysis examined how change in living arrangement, in-person interaction outside the household, quality of relationship with family and friends, and perceived social support affected trajectories of isolation stress, COVID worry and sadness.

**Results:**

While their living arrangements (Odds Ratio [OR] = 0.95, 95% Confidence Interval [CI] = 0.87, 1.03) and relationship quality (OR = 0.94, 95% CI = 0.82, 1.01) remained stable, older adults experienced fluctuations in perceived social support (linear Slope b = -1.42, s.e. = 0.16, *p* < .001, quadratic slope b = 0.50, s.e. = 0.08, *p* < .001, cubic slope b = -0.04, s.e. = 0.01, *p* < .001) and increases in in-person conversations outside the household (OR = 1.19, 95% CI = 1.09, 1.29). Living with a spouse/partner stabilized isolation stress (change in linear slope b = 1.16, s.e. = 0.48, *p* < .05, in quadratic slope b = -0.62, s.e. = 0.26, *p* < .05, and in cubic slope = 0.09, s.e. = 0.04, *p* < .05) and COVID worry (change in quadratic slope b = -0.66, s.e. = 0.32, *p* < .05 and in cubic slope = 0.09, s.e. = 0.04, *p* < .05) over time. Individuals with better relationship quality with friends had decreased sadness over time (OR = 0.90, 95% CI = 0.82, 0.99). Changes in social support were associated with greater fluctuations in isolation stress and COVID worry.

**Conclusions:**

During the pandemic, social interactions are protective and lack of stability in feeling supported makes older adults vulnerable to stress. Efforts should focus on (re)building and maintaining companionship and support to mitigate the pandemic’s negative impact.

**Supplementary Information:**

The online version contains supplementary material available at 10.1186/s12889-022-14103-x.

## Background

The COVID-19 pandemic poses significant challenges globally, threatening the lives and livelihoods of millions of people. The mental health consequence of the pandemic is clear. Across Asia and Europe, population-based studies show elevated stress, depression, and loneliness, as well as lower levels of self-esteem and life satisfaction in multiple countries [1–4]. Amidst historical levels of death, grief, worry, quarantine fatigue, and economic uncertainty, Americans reported the lowest level of happiness in nearly fifty years [5]. Estimates from polls and census data suggest that up to forty percent adults in the United States experienced symptoms of depression or anxiety in 2020 [6, 7]. One primary reason for impaired psychological well-being is social isolation and loneliness introduced by lockdowns, stay-at-home orders, and social distancing measures [8, 9].

In this paper, we focus on life under stay-at-home orders among older adults in the US during the COVID-19 pandemic. Beginning March 2020, a number of US states issued stay-at- home or safer-at-home orders. By May 2020, almost all states ordered nonessential businesses to close and prohibited gatherings. Individuals who were not considered essential employees were asked not to leave their homes apart from medical care or grocery shopping. During the summer of 2020, most states began reopening, but quickly re-implemented the restrictions in response to large spikes of COVID cases in the fall.

Older adults are particularly vulnerable to the prolonged social isolation and limited social interaction during the pandemic for two reasons. First, while the COVID-19 pandemic has forced acute social isolation for everyone, older adults may experience a greater reduction in social interaction and support, due to age-based patterns in health behaviors, labor force exits and mortality. Compared to their younger counterparts, older adults are more likely to engage in mitigation practices such as maintaining physical distance, avoiding crowded places and restaurants, and cancelling social and recreational activities [10]. The percentage of older workers leaving the workforce rose sharply to 32% in 2020, from about 15% before the pandemic [11]. Unlike those during mid-career, labor force exits close to retirement age are often permanent [12], likely resulting in the contraction of personal networks and reduced support [13]. Loss of spouse, siblings and lifelong friends are common among older adults, as the pandemic’s death toll has been largest in this age group [14], thereby disrupting their care and support systems. Voluntary or not, limited social interaction and reduced support can be a direct result of the pandemic. For older adults, these can be significant acute stressors and negatively impact their emotional wellbeing and mental health [15–19].

Second, the COVID-19 pandemic aside, older adults already face challenges staying socially connected and may not benefit as much from the protective effects of social relationships. Social networks and the support derived from them play a critical role in maintaining socioemotional well-being over the life course [20–23]. However, physical functional limitations [24], death of spouse, kin and friends [25, 26], and limited cross-age interaction in personal networks [27] are common during older adulthood. These challenges can introduce significant and often deleterious changes in the structure and quality of social relationships for older adults and subsequently in health and longevity [28–33]. Coping with bereavement and health problems requires even more companionship and support, the absence of which may then lead to deteriorating emotional well-being [25, 34]. In this context, the pandemic’s mental health impact on older adults is significant, largely because the pervasive and chronic social disconnectedness that existed long before COVID in older adults makes these individuals more vulnerable given limited access to the support needed to cope with pandemic stress.

Although prior research provides support for the basic prediction of worse mental health in older adults as a result of the pandemic, it is uncertain if such a prediction represents the experience of older adults over an extend period of time. This is especially important given the context. After the initial wave of infections, a number of U.S. states quickly lifted or modified existing stay-at-home orders, but repeated outbreaks and emerging variants generated substantial heterogeneity in not only COVID trends and cases, but social distancing measures across states. Moreover, even within states, social distancing measures are being constantly updated. In light of this rapidly evolving social context, one may hypothesize fluctuations in mental wellbeing among older adults. Alternatively, the constant changes brought by the pandemic and social distancing measures can result in a feeling of uncertainty for older adults, which in turn lead to persistent elevated stress levels. We may therefore observe worse, but stable mental wellbeing over time. Presently, there is a clear need to characterize change in mental wellbeing over an extended period of time during the COVID pandemic and identify predictors that may be associated with the change. We do not know, for example, whether social support stabilizes mental wellbeing, or exacerbates the fluctuations. To date, however, most of our knowledge about the pandemic’s mental health impact come from cross-sectional studies [2, 8, 15, 35], short-term observations over a few weeks at the beginning of the pandemic [1, 16, 36–38], or before vs. during pandemic comparisons with limited follow-ups [3, 18, 19]. Repeated assessments of social interaction and mental health over longer periods of time are not widely available.

The current study aims to address this issue by characterizing change in social interaction over six months during the pandemic and its impact on emotional wellbeing in older Americans. We take advantage of data from a panel study conducted May through October 2020 to track changes in current living arrangements, in-person interaction outside the household, quality of relationship with family and friends, and perceived social support and examine how stability and change in social interaction affect pandemic-specific emotional health indicators, including isolation stress and COVID worry, as well as general sadness.

## Methods

### Study design and participants

Data came from a larger panel study with six repeated assessments of social interaction and emotional wellbeing conducted monthly from May to October 2020. The purpose of the study was to evaluate the mental health impact of social distancing guidelines imposed as part of the public health measures to limit the spread of COVID-19. Therefore, participant recruitment was limited to fourteen US states with active stay-at-home orders in May 2020, as residents in these areas were more likely to experience social isolation due to the restrictions. The states represented in our study were: Alabama, California, Oregon, Kentucky, Louisiana, Maine, Maryland, New Jersey, New York, Pennsylvania, Tennessee, Virginia, Washington, and Washington DC. Blacks, Hispanics, and individuals who had low household income (defined as less than $30,000 a year) were oversampled, since the pandemic disproportionately affected racial and ethnic minorities and the poor [39–41].

**Fig. 1:**
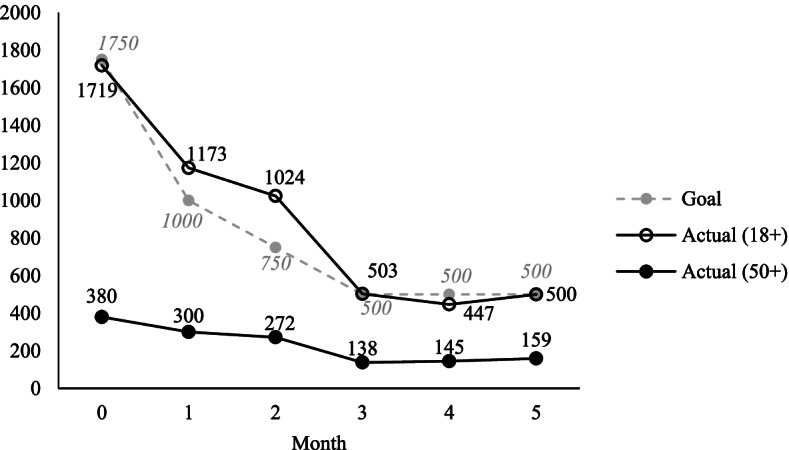
Number of participants retained in the study over time

For the current report, we drew from the original study a subsample of adults aged 50 and over. As shown in Fig. 1, from May 5 through May 26, 2020, a total of 380 participants aged 50 and over were enrolled in the study. They completed an online baseline (Month 0) survey via Qualtrics. The original study by design introduced panel attrition due to funding restrictions. At each follow-up, only the participants who had responded to the previous survey were reinvited and recruitment was closed upon reaching enrollment. As a result, the number of older adult participants in the current analytic sample was 300 at Month 1, 272 at Month 2, 138 at Month 3, 145 at Month 4, and 159 at Month 5. Compared to their younger counterparts, older participants in the study were less likely to have an incomplete panel (i.e., missing one or more follow ups, 81% versus 88%, *p* < . 001) and on average completed more follow ups (3.67 versus 2.97, *p* < 0.001). These participants contributed a total of 1,394 repeated observations over the course of the study for analysis. The IRB at University of Pennsylvania determined that the study was exempt from review. All research was performed in accordance with relevant guidelines and regulations. Online written consent was obtained from all participants prior to beginning the survey.

### Measurements of social interaction

We included both pandemic-specific (in-person interaction) and general (living arrangement, relationship quality and social support) metrics of social interaction. For living arrangements, we asked participants to first indicate how many people currently live in their home (excluding self) and then to specify their relationship to each of the persons in their home. Two dichotomized variables were created to indicate living arrangement—*living alone* (= 1, otherwise = 0) and *living with spouse or partner* (= 1, otherwise = 0). To measure *in-person interaction*, we asked them to indicate how many people from outside the household they had an in-person conversation with during the past month. Response categories include none (= 0), 1–2 per week (= 1), 3–4 per week (= 2), 5–6 per week (= 3) and 7 or more per week (= 4).

Family relationships and friendships were assessed separately. The participants were first asked how the *quality of relationships with family members* had changed during the past month and second, how the *quality of relationships with friends* changed during the past month. For each question, response categories included a lot worse (= -2), a little worse (= -1), about the same (= 0), a little better (= 1), and a lot better (= 2). *Perceived social support* was measured with an adapted version of the six-item brief version of the Perceived Social Support Questionnaire [42]: (1) I experience a lot of understanding and security from others; (2) I know a very close person whose help I can always count on; (3) If necessary, I can easily borrow something I might need from neighbors or friends; (4) I know several people with whom I like to do things; (5) When I am sick, I can without hesitation ask friends and family to take care of important matters for me; (6) If I am down, I know to whom I can go without hesitation. The original response categories for each item included strongly disagree, disagree, neutral, agree, and strongly agree and they were dichotomized (strongly agree = 1, otherwise = 0) due to a ceiling effect. Internal consistency of the recoded items ranged from 0.78 to 0.87 across months and a summed score of six dichotomized items were created.

Living arrangement, in-person interaction, and relationship quality were time-varying and measured consistently across six monthly assessments. Perceived social support was not measured at Month 2 due to limits in survey length. We used estimated trajectory of social support to impute values at Month 2 when examining its relationship with emotional wellbeing (see Analysis Plan below).

### Measurements of emotional wellbeing

Emotional wellbeing during the pandemic was measured by isolation stress, COVID worry, and sadness. All three variables were time-varying and measured consistently across six monthly assessments. *Isolation stress* was measured by four items asking participants to evaluate the impact of pandemic-related physical restrictions [43]. Specifically, they were asked to indicate on a 5-point rating scale ranging from not at all (= 0) to extremely (= 4) for the following: During the past month. (1) how stressful have the restrictions on leaving home been for you? (2) how stressful have changes in family contacts been for you? (3) how stressful have changes in social contacts been for you? (4) how much has cancellation of important events (such as graduation, prom, vacation, etc.) in your life been difficult for you? Given good internal consistency of the items in the current analytic sample across monthly assessments (α’s = 0.77 to 0.82), a summed score was created to indicate overall level of isolation stress the participant experienced during the past month.

*COVID worry* was measured by a summed score of five items designed for this study assessing an individual’s disease worry, risk perception, and perceived controllability in the context of COVID-19: During the past month. (1) how worried have you been about being infected? (2) how worried have you been about friends or family being infected? (3) how worried have you been about your physical health being influenced by Coronavirus/COVID-19? (4) how worried have you been about your mental/emotional health being influenced by Coronavirus/COVID-19? (5) how much are you reading or talking about Coronavirus/COVID-19? For the first four items, response categories included not at all (= 0), slightly (= 1), moderately (= 2), very (= 3) and extremely (= 4) and for the last item response categories included never (= 0), rarely (= 1), occasionally (= 2), often (= 3) and most of the time (= 4). Internal consistency of the items was high across monthly assessments (α’s = 0.78 to 0.86).

Finally, *sadness* was measured by asking the participants to indicate how happy versus sad they were during the past month. Response categories include very happy/cheerful (= -2), moderately happy/cheerful (= -1), neutral (= 0), moderately sad/depressed/unhappy (= 1), and very sad/depressed/unhappy (= 2).

### Measurement of covariates

We measured the participants’ age at baseline in years. Gender was measured by a dichotomized variable indicating female (= 1, otherwise = 0). Race and ethnicity were captured by three dichotomized variables indicating non-Hispanic White (= 1, otherwise = 0; reference group), non-Hispanic Black (= 1, otherwise = 0), and Hispanic (= 1, otherwise = 0). Education was measured by an ordinal variable indicating highest level of education completed, which included less than high school (= 0), high school diploma or GED (= 1), some college or 2-year degree (= 2), 4-year college graduate (= 3), and more than college (= 4). Participants reported on their annual household income by selecting one of the following categories: less than $30,000, $30,000 to $49,999, $50,000 to $74,999, $75,000 to $99,999 $100,000 to $249,999, more than $250,000. A dichotomized variable indicating low income was used in all multivariate analyses (< $30,000 = 1; otherwise = 0). We also noted if the participant was currently working for pay (= 1, otherwise = 0). Because enrollment was capped at a lower number at Month 3 through Month 5, we adjusted for potential panel attrition bias with a dichotomized indicator of incomplete panel (i.e., completed fewer than six monthly assessments) included in all models as a covariate. In preliminary analyses (not shown), we compared this method to a correction instrument calculated from a two-stage Heckman selection bias model [44] and found the two methods do not produce substantively different results. We opted to use a dichotomized indicator to adjust for panel attrition in the final models as this method did not rely on accurate specification of the selection bias, which was difficult to ascertain in a panel study spanning over a relatively short period of time.

### Statistical model

To examine intra-individual change in social interaction and emotional wellbeing across repeated observations and the associations between them, we used random-intercept logistic regression models to estimate binary outcomes (living alone and living with spouse or partner), random-coefficient proportional odds models to estimate ordinal outcomes (in-person interaction, quality of relationship with family and with friends, and sadness), and random-coefficient growth curve models with a Gaussian link to estimate continuous outcomes (social support, isolation stress, and COVID worry). These random effects models explicitly model longitudinal dependency with individual-specific trajectories of change. All models used month as the time variable. Since change over time could be nonlinear, we tested in preliminary analysis linear, quadratic, cubic, and quartic specifications of time. Best fitting model specification for each outcome was determined by statistical significance of the coefficient(s) and Bayesian Information Criterion (BIC) and used for final analysis. Specifically, we modeled change in living arrangement, in-person interaction, quality of relationship with family and with friends, and sadness as linear function of time and change in perceived social support, isolation stress, COVID worry as a cubic function of time.

### Analysis plan

The analysis proceeded in three stages. First, we described the sample with regards to sociodemographic characteristics and panel attrition. Second, we described change in social interaction and emotional wellbeing over six months during the pandemic, by estimating time-based trajectories of the variables without adjusting for covariates. From these models we extracted estimated probabilities (for binary or ordinal variables) or values (for continuous variables) and plotted them over time to visualize the time trend. Third, we examined the associations between social interaction variables, as time-varying covariates, and time-based trajectories of emotional wellbeing, adjusting for covariates. Estimated values of perceived social support were extracted from the trajectory model in stage 2 and used to impute social support at Month 2 in models estimating trajectories of emotional wellbeing. Due to difficulty interpreting an interaction between a continuous time-varying covariate and time, we created a dichotomized variable representing higher levels of perceived social support (sum social support score = 6) to examine how social support affected the rates of change in trajectories of emotional wellbeing. In preliminary analysis, we tested interactions between social interaction variables and time, to determine how each social interaction variable affected the trajectory of change in emotional wellbeing. Only statistically significant interactions were retained in the final models and presented in the results.

## Results

### Sample characteristics

On average, participants in the sample were 57 years of age in May 2020 (SD = 5.52; see Table 1), with the majority of them identifying as female. Half of the participants were non-Hispanic White, about one-third were non-Hispanic Black and slightly less than one-fifth were Hispanic. Few participants had less than high school education. About 15% of the participants completed high school, about 40% completed some college, a quarter were 4-year college graduates and over one-fifth had schooling beyond college. A third had less than $30,000 in annual household income and about 61% were working for pay in May 2020. Over the course of the study, less than one-fifth of the participants completed all six repeated assessments and the rest had an incomplete panel. On average, the number of repeated assessments completed was 3.67 (SD = 1.80). Preliminary analysis (not shown) revealed that Blacks, Hispanics, and individuals with low income were more likely to drop out of the panel and completed fewer assessments than their White and higher income counterparts, respectively. Women were more likely to complete the entirety of the study than did men.

**Table 1:** Sample characteristics at baseline

Variable	Mean (SD) or Proportion
Age	57.24(5.52)
Gender	
Male	45%
Female	55%
Race and Ethnicity	
Non-Hispanic White	49%
Non-Hispanic Black	32%
Hispanic	18%
Education	
Less Than High School	1%
High School Diploma or GED	15%
Some College or 2-Year Degree	40%
4-Year College	24%
More Than College	21%
Annual Household Income	
Less Than $30,000	33%
$30,000—$49,999	15%
$50,000—$74,999	17%
$75,000- $99,999	15%
$100,000—$249,999	19%
$250,000 or More	1%
Currently Working for Pay	61%
Incomplete Panel	81%
Number of Assessments Completed	3.67 (1.80)

### Stability and change in social interaction and emotional wellbeing during the pandemic

**Fig. 2:**
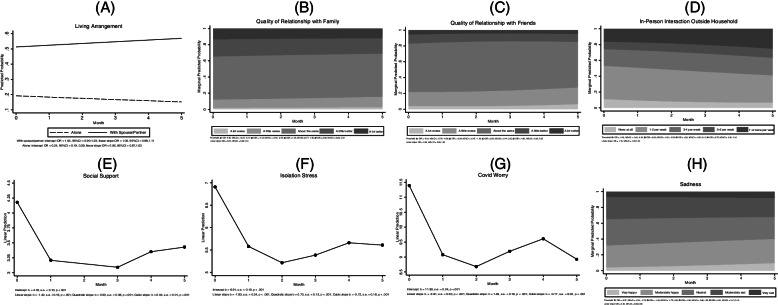
Change in social interaction and emotional wellbeing may 2020 (baseline; month 0) through october 2020 (month 5)

We observed both stability and change in social interaction and emotional wellbeing in older adults over the six-month period (see Fig. 2 for results). Slightly more than half of the participants lived with a partner and less than one-fifth lived alone at the beginning of the study (Panel A, Fig. 2). Model estimates suggested essentially no change in living arrangement over the course of the study. Similarly, quality of family relationship (Panel B, Fig. 1) and friendship (Panel C, Fig. 2) remained stable over time. There was a modest linear increase in the number of people outside the household with whom participants had in-person conversations (Panel D, Fig. 2). Over six months, the estimated probability of talking to seven or more people outside the household increased substantially whereas the probability for none or infrequent in-person interaction outside the home decreased considerably. The trajectory of social support was best characterized by a cubic function. From baseline to Month 1, perceived social support dropped by about a unit, remained relatively low from Month 1 to Month 3, and slightly increased after Month 3 (Panel E, Fig. 2).

Turning to emotional wellbeing, we found that isolation stress followed a cubic trajectory: from baseline to Month 1, isolation stress had a rapid decrease of almost two units by Month 2, and a modest increase of half of a unit from Month 2 to Month 4 before plateauing from Month 4 to Month 5 (Panel F, Fig. 2). COVID worry was also best characterized by a cubic trajectory (Panel G, Fig. 2). There was a sizable drop in COVID worry during the first month of the study, albeit more muted than what was observed in isolation stress. After Month 2, COVID worry largely fluctuated. In contrast, sadness had a steady linear decrease over time (Panel H, Fig. 2). Each month was associated with a lower chance of being in a more sad or depressed category.

### The protective effect of social interaction on emotional wellbeing

**Fig. 3:**
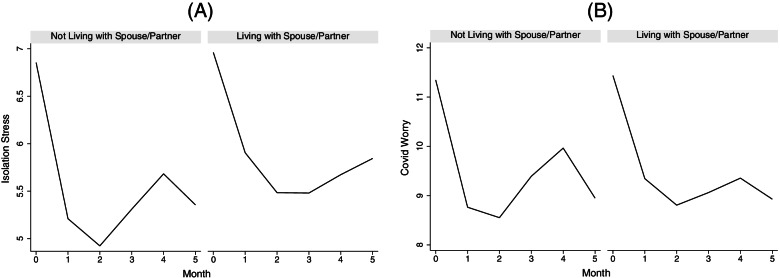
Living with spouse or partner stabilizes isolation stress and COVID worry over time

Next, we examined how social interaction variables, as time-varying covariates, were associated with trajectories of emotional wellbeing in older adults (see Table 2 for results). Living alone was associated with lower initial levels (i.e., intercept) of isolation stress (b = -0.97, s.e. = 0.47, *p* < 0.05) but did not affect the rates of change (i.e., slopes). Living alone also had no effect on COVID worry or the sadness. Living with spouse or partner was associated with rates of change in isolation stress over time. Specifically, isolation stress followed a cubic trajectory over time and living with spouse or partner moderated the trajectory, by counterbalancing the rapid linear decline (b = 1.16, s.e. = 0.48, *p* < 0.05), slowing the quadratic acceleration (b = -0.62, s.e. = 0.26, *p* < 0.05), and offsetting the cubic reduction in acceleration (b = 0.09, s.e. = 0.04, *p* < 0.05) in the trajectory of isolation stress. In other words, compared to those who did not live with a spouse or partner during the pandemic, older adults living with spouse or partner exhibited more stability in isolation stress over time (Panel A, Fig. 3). A similar stabilizing effect of living with spouse or partner was observed, albeit more muted, for the trajectory of COVID worry (Table 2 and Panel B, Fig. 3).

**Table 2:** The Associations Between Social Interaction and Emotional Wellbeing Among Adults Aged 50 and Over May Through October 2020

	Isolation stress	COVID Worry	Sadness
	b(s.e.)^Sig^	b(s.e.)^Sig^	OR[95% CI]^Sig^
Intercept	9.43(2.00)***	9.94(2.76)***	
Thresholds:			
${\kappa }_{1}$			-6.35 [-9.78, -2.93]***
${\kappa }_{2}$			-2.50[-2.73, 0.13]
${\kappa }_{3}$			0.14[-3.26, 3.53]
${\kappa }_{4}$			3.59[0.17, 7.00]*
Time	-2.22(0.38)***	-3.91(0.48)***	0.89[0.82, 0.96]**
Time^2^	0.91(0.21)***	1.72(0.26)***	
Time^3^	-0.10(0.03)***	-0.20(0.04)***	
Living Arrangement			
Alone	-0.97(0.47)*	-0.53(0.63)	0.86[0.39,1.91]
With Spouse/Partner	-0.59(0.44)	-0.11(0.58)	0.56[0.29,1.11]
With Spouse/Partner × Time	1.16(0.48)*	1.05(0.60)	
With Spouse/Partner × Time^2^	-0.62(0.26)*	-0.66(0.32)*	
With Spouse/Partner × Time^3^	0.09(0.04)*	0.09(0.04)*	
Quality of Relationship			
With Family	-0.36(0.10)***	0.14(0.12)	0.74[0.62,0.90]**
With Friends	-0.46(0.11)***	-0.01(0.14)	0.77[0.59,1.01]
With Friends × Time			0.90[0.82,0.99]*
With Friendst × Time^2^			
With Friends × Time^3^			
In-Person Interaction	-0.06(0.07)	0.004(0.09)	0.88[0.77,0.99]*
High Social Support	0.59(0.28)*	0.26(0.35)	0.85[0.60, 1.20]
High Social Support × Time	-1.69(0.63)**	-2.23(0.79)**	
High Social Support × Time^2^	0.58(0.33)	0.79(0.41)	
High Social Support × Time^3^	-0.06(0.04)	-0.08(0.05)	
Age	-0.04(0.03)	0.02(0.04)	0.98[0.93, 1.04]
Female	0.24(0.34)	0.34(0.47)	1.17[0.66, 2.09]
Race and Ethnicity:			
White (reference)	–	–	–
Black	0.10(0.40)	0.45(0.55)	0.47[0.24, 0.93]*
Hispanic	0.51(0.48)	0.60(0.66)	1.49[0.66, 3.34]
Education	-0.07(0.18)	-0.01(0.24)	1.02[0.50, 2.02]
Low Income	-0.75(0.41)	-0.04(0.57)	1.00[0.50, 2.02]
Currently Working	0.23(0.23)	-0.04(0.30)	0.99[0.65, 1.51]
Incomplete Panel	0.11(0.42)	-0.21(0.59)	2.40[1.18, 4.90]*

^*^p < .05 **p < .01 ***p < .001

**Fig. 4:**
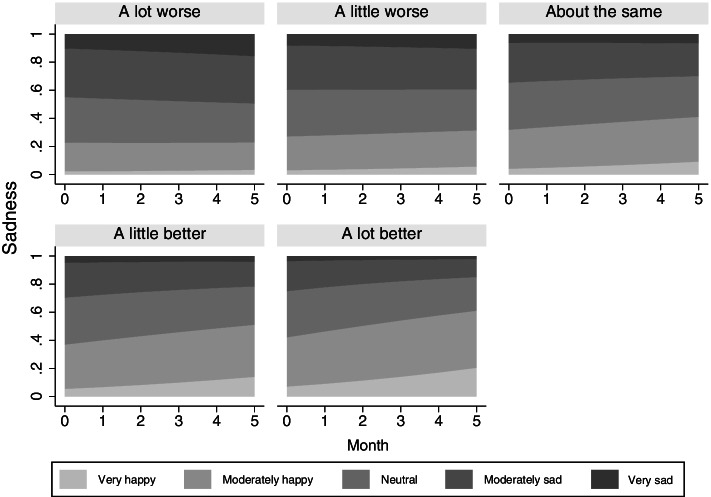
Better friendship quality reduces sadness over time

**Fig. 5:**
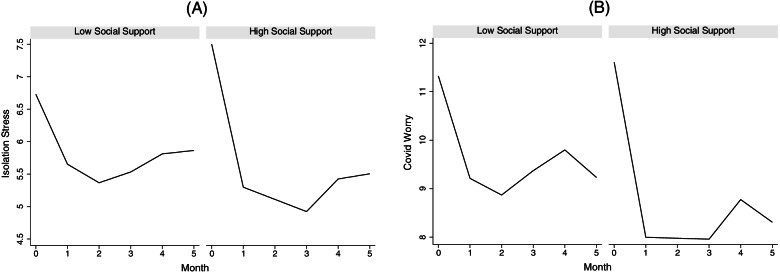
Social support is associated with fluctuations in on isolation stress and COVID worry over time

Older adults with stronger family relationships had lower levels of isolation stress (b = -0.36, s.e. = 0.10, *p* < 0.001) and were less likely to be more sad or depressed (Odds Ratio [OR] = 0.74, 95% Confidence Internal [CI] = 0.62, 0.90). Quality of family relationship did not moderate the rates of change in isolation stress or sadness. Quality of friendships was associated with lower initial levels of isolation stress (b = -0.46, s.e. = 0.11, *p* < 0.001) and moderated the trajectory of sadness, by amplifying the linear reduction in sadness over time (OR = 0.90, 95% CI = 0.82, 0.99). The estimates indicate that each unit increase in quality of friendships was associated with about 20% reduction in the odds of being in a more sad/depressed category. For older adults whose relationships with friends were a little or a lot better, the probability for reporting moderately to very happy increased noticeably whereas a decrease was observed in reporting moderately to very sad (Fig. 4). The COVID worry trajectory were not affected by the quality of family relationships and friendships. While in-person interaction did not influence emotional wellbeing, social support was associated with isolation stress and COVID worry. Interestingly, high social support was associated with higher initial level of isolation stress (b = 0.59, s.e. = 0.28, *p* < 0.05). During the first two months, however, older adults with high social support had a much more rapid decline in isolation stress (b = -1.69, s.e. = 0.63, *p* < 0.01) and in COVID worry (b = -2.23, s.e. = 0.79, *p* < 0.01). After Month 3, individuals with high social support did not differ from those with lower levels of social support with regards to change in isolation stress. Overall, higher social support appeared to have resulted in greater fluctuations over time in emotional wellbeing (Fig. 5).

## Discussion

This study examines how social interaction affects emotional wellbeing in older Americans during a time of imposed social distancing due to the COVID-19 pandemic. The individual approaches taken by state and local governments in the US may have impacted social ties in different ways. The panel design of our study has permitted a characterization of change in both pandemic specific and general metrics of social interaction and emotional wellbeing while the social context varied and was rapidly evolving.

Longitudinal data collected at the beginning of the pandemic generally show an uptick in mental health issues in the population under study [1, 3, 6, 37]. Our results suggest stability and change over time across social interaction and emotional wellbeing outcomes, the pattern of which are largely consistent with how the pandemic evolved in the US May through October 2020. For example, we observe a significant drop in isolation stress and COVID worry in the first two months (May through June 2020), likely because early lockdowns resulted in low case numbers in the summer of 2020, leading to the belief that the pandemic would soon come to an end and social distancing restrictions were being lifted. The fluctuations in isolation stress and COVID worry in later months are in line with the spike of cases and re-implementation of social distancing measures. We however did not see a similar pattern of fluctuation in in-person interaction, which was a pandemic-specific metric of social interaction. Over time, older adults in our sample increased in-person conversations outside the household, despite the restrictions and increasing COVID cases in the fall of 2020. Therefore, the association between in-person interaction and emotional wellbeing is weakening, as the pandemic transitioned from being an acute stressor to a chronic stressor. The literature has called for developing interventions to maintain social interaction during the pandemic [8, 9], perhaps with the aid of technology [35]. Our finding however suggests that merely increasing social interactions may not effectively serve older adults’ emotional needs.

For general social interaction metrics, our results suggest that living arrangement and quality of relationships stayed stable over the six-month period whereas perceived social support fluctuated, exhibiting a trajectory similar to isolation stress and COVID worry. In contrast to the expected stability in relationship quality, this fluctuation in social support is surprising. Relationship quality and social support are often positively correlated because higher quality relationships tend to be the ties that are able and willing to lend support [45]. This decoupling of the two metrics may itself be due to the pandemic. Social support indicates the exchange of resources from one person to another [21]. In response to the pandemic, individuals worldwide face heightened levels of stress and daily stressors [2, 3, 5, 8]. Thus, they may simply have fewer resources leftover to lend to another after coping with their own stress. Similarly, previous research has reported that the closure of social support services during COVID is detrimental to older adults who rely on them as the primary source of social interaction and integration [46].

Perhaps because of the differences in mutability, we found that relationship quality and social support were associated with emotional wellbeing in different ways. Results suggest that better family relationships and friendships serve as a protective buffer against COVID worry and sadness—a finding that has been reported in samples in the U.S. [35], the U.K. [47], and Italy [3]. Perceived social support, however, was associated with greater isolation stress at the beginning of the observation period. There are several possible explanations for this pattern. First, perceived social support tends to be correlate with network size especially in small personal networks [48], with individuals reporting higher levels of social support also having more social ties. With a larger network, one may receive more information about COVID and have more conversations about COVID. As COVID-related topics are repeatedly discussed and COVID worries are shared within the network, existing stress and worry may increase [38, 49]. This may especially be the case at the beginning of the pandemic when there was a great amount of confusion, uncertainty, and anxiety in public messages about COVID. Second, research has long suggested that social support and social ties influence mental health via the mechanisms of integration and feeling of connectedness and attachment [50]. It stands to reason that social support may be particularly salient to older adults who have more ties and support, because they value such connectedness more than those reporting lower levels of support. As a result, pandemic-imposed social isolation may be particularly detrimental to these individuals because of the centrality of social support in their lives. Finally, since we observe a very similar pattern of fluctuation in social support, isolation stress, and COVID worry, the association between social support and emotional wellbeing may be spurious or due to reverse causality. It is possible that the pandemic directly affects older adults by increasing stress and worry while simultaneously reducing perceived support. Alternatively, individuals who are under stress and worry excessively can feel particularly unsupported [51].

After the first two months, older adults with higher social support reported more rapid decreases in stress and worry, in line with the stress-buffering effect of social support. One explanation for this rebound is that participants gradually adjust to life under stay-at-home orders over time, and as they adjust, they strive to rebuild or maintain support. Taken together, the findings suggest that fluctuations in the perceived support may result in greater fluctuations in emotional wellbeing for older adults. Prior research has shown that programs such as peer support groups and befriending services may help address social isolation among older adults [46]. Thus, future behavioral and policy-related interventions should seek to preserve and enhance the resources available to older adults to alleviate these oscillations in perceived support. At the same time, public-wide remarks regarding the pandemic should acknowledge the importance of social support when communicating oncoming challenges and the potential impact of those challenges on emotional wellbeing. For example, the messages could provide strategies for safe social interactions, particularly for populations such as older adults who already experience deficits in social interactions pre-pandemic.

Results regarding living arrangement revealed additional ways through which social interaction affected emotional wellbeing. Previous research has pointed out that living with others during the pandemic can have mental health benefits [37]. While we did not find a direct effect, living with spouse or partner was found to stabilize change in emotional wellbeing measures over time. Since there is little change in older adults’ living arrangement over the study period, this result suggests that one’s spouse or partner can be a stable source of companionship and support, thereby protecting individuals from recurrent episodes of stress associated with the pandemic. Living alone is associated with lower levels of isolation stress. This is likely a pandemic specific finding, as older adults living alone prior to the pandemic may already be experiencing high levels of isolation and disconnectedness and are, therefore, less sensitive to the effect of pandemic-imposed social distancing measures. Alternatively, compared to living arrangement, other aspects of social connectedness such as relationship quality may have a larger impact on older adults’ emotional wellbeing. Living alone may not necessarily lead to loneliness if older adults benefit from supportive networks and high-quality relationships [52].

Our study has several limitations. First, our participants are from fourteen US states and the sample is nonrepresentative of the U.S. population aged 50 and over. Because the survey was administered online, older adults who lack access to the internet and/or technology are likely underrepresented in our study. Future research should address similar questions in larger scale studies of representative samples of older adults both in the US and other countries, and take into account how the method of data collection may bias sampling and the interpretation of results. Second, due to funding restrictions, panel attrition was introduced by design. While we adjusted for having an incomplete panel, residual non-random panel attrition may still bias estimates. Third, because of survey burden concerns, our study included limited measurements of the broader social context, some of our assessments were brief, and a key variable, social support, was not assessed at Month 2. It will be important for future research to include measures with superior psychometric properties and account for additional confounding factors that may be affect the relationship between social interaction and emotional health. Finally, while we found that living arrangement was associated with emotional wellbeing during pandemic, our analysis is not sufficiently powered to compare different types of living arrangement. A six-month observation window may also be too short to capture the changes. Future research should better characterize pandemic-induced change in living arrangement in older adults with appropriate designs.

## Conclusions

Despite the limitations, this study makes contributions to the literature by analyzing co-occurring changes of social interaction and emotional wellbeing longitudinally and by showing how each metric of social interaction differentially affects older adult’s emotional wellbeing. While overall the results suggest that social interaction is protective, there is a lack of stability in feeling supported during the pandemic, which in turn makes older adults vulnerable to stress. Thus, our study offers novel insights into *how* social interaction may be associated with mental health. Policy and public health interventions should focus on (re)building support and companionship to bolster psychological well-being in older adults.

## Supplementary Information

**Additional file 1.**

## Data Availability

The dataset supporting the conclusions of this study are included as a supplementary file.

## References

[1] O'Connor RC, Wetherall K, Cleare S, McClelland H, Melson AJ, Niedzwiedz CL, O'Carroll RE, O'Connor DB, Platt S, Scowcroft E, Watson B (2021). Mental health and well-being during the COVID-19 pandemic: longitudinal analyses of adults in the UK COVID-19 Mental Health & Wellbeing study. Br J Psychiatry.

[2] Pancani L, Marinucci M, Aureli N, Riva P (2021). Forced social isolation and mental health: a study on 1,006 Italians under COVID-19 lockdown. Front Psychol.

[3] Pierce M, Hope H, Ford T, Hatch S, Hotopf M, John A, Kontopantelis E, Webb R, Wessely S, McManus S, Abel KM (2020). Mental health before and during the COVID-19 pandemic: a longitudinal probability sample survey of the UK population. Lancet Psychiatry.

[4] Wang C, Pan R, Wan X, Tan Y, Xu L, McIntyre RS, Choo FN, Tran B, Ho R, Sharma VK, Ho C (2020). A longitudinal study on the mental health of general population during the COVID-19 epidemic in China. Brain Behav Immun.

[5] NORC. Historic shift in Americans’ happiness amid pandemic. 2020. https://www.norc.org/PDFs/COVID%20Response%20Tracking%20Study/Historic%20Shift%20in%20Americans%20Happiness%20Amid%20Pandemic.pdf. Accessed 1 Jan 2022.

[6] Eichstaedt JC, Sherman GT, Giorgi S, Roberts SO, Reynolds ME, Ungar LH, Guntuku SC (2021). The emotional and mental health impact of the murder of George Floyd on the US population. Proc Natl Acad Sci.

[7] Panchal N, Kamal R, Orgera K, Cox C, Garfield R, Hamel L, Chidambaram P. The implications of COVID-19 for mental health and substance use. 2020*.* https://www.kff.org/coronavirus-COVID-19/issue-brief/the-implications-of-COVID-19-for-mental-health-and-substance-use//. Accessed 1 Jan 2022.

[8] Wang C, Tee M, Roy AE, Fardin MA, Srichokchatchawan W, Habib HA, Tran BX, Hussain S, Hoang MT, Le XT, Ma W (2021). The impact of COVID-19 pandemic on physical and mental health of Asians: A study of seven middle-income countries in Asia. PLoS ONE.

[9] Williams CY, Townson AT, Kapur M, Ferreira AF, Nunn R, Galante J, Phillips V, Gentry S, Usher-Smith JA (2021). Interventions to reduce social isolation and loneliness during COVID-19 physical distancing measures: A rapid systematic review. PLoS ONE.

[10] Hutchins HJ, Wolff B, Leeb R, Ko JY, Odom E, Willey J, Friedman A, Bitsko RH (2020). COVID-19 mitigation behaviors by age group—United States, April–June 2020. Morb Mortal Wkly Rep.

[11] Quinby L, Rutledge MS, Wettstein G. How Has COVID-19 Affected the Labor Force Participation of Older Workers?. Center for Retirement Research at Boston College Working Papers 2021-13. Accessed on Jan 1, 2021 https://crr.bc.edu/working-papers/how-has-covid-19-affected-the-labor-force-participation-of-older-workers/#:~:text=Among%20workers%20ages%2055%20and,remote%20work%20saw%20disproportionate%20impacts.

[12] Chan S, Huff SA (2001). Job loss and employment patterns of older workers. J Law Econ.

[13] Kauppi M, Virtanen M, Pentti J, Aalto V, Kivimäki M, Vahtera J, Stenholm S (2021). Social network ties before and after retirement: a cohort study. Eur J Ageing.

[14] Verdery AM, Smith-Greenaway E, Margolis R, Daw J (2020). Tracking the reach of COVID-19 kin loss with a bereavement multiplier applied to the United States. Proc Natl Acad Sci.

[15] Birditt KS, Turkelson A, Fingerman KL, Polenick CA, Oya A (2021). Age differences in stress, life changes, and social ties during the COVID-19 pandemic: Implications for psychological well-being. Gerontologist.

[16] Klaiber P, Wen JH, DeLongis A, Sin NL (2021). The ups and downs of daily life during COVID-19: Age differences in affect, stress, and positive events. J Gerontol: Series B.

[17] Kim HH, Jung JH (2021). Social isolation and psychological distress during the COVID-19 pandemic: A cross-national analysis. Gerontologist.

[18] Peng S, Roth AR. Social isolation and loneliness before and during the COVID-19 pandemic: a longitudinal study of US adults older than 50. J Gerontol B. 2022;77(7):e185–e190. 10.1093/geronb/gbab068 PMC8083229 33870414

[19] Van Tilburg TG, Steinmetz S, Stolte E, van der Roest H, de Vries DH (2021). Loneliness and mental health during the COVID-19 pandemic: A study among Dutch older adults. J Gerontol B.

[20] Antonucci TC, Ajrouch KJ, Birditt KS (2014). The convoy model: Explaining social relations from a multidisciplinary perspective. Gerontologist.

[21] Cohen SE, Syme SI. Social support and health. Orlando: Academic Press; 1985.

[22] House JS, Landis KR, Umberson D (1988). Social relationships and health. Science.

[23] Kawachi I, Berkman LF (2001). Social ties and mental health. J Urban Health.

[24] Schafer MH (2018). (Where) is functional decline isolating? Disordered environments and the onset of disability. J Health Soc Behav.

[25] Brown SL, Mellencamp KA, Lin IF. Sole family survivors: older adults lacking family of origin kin. J Gerontol B. 2022;77(5):930–35. 10.1093/geronb/gbab239 PMC9071394 34969095

[26] Verdery AM, Margolis R (2017). Projections of white and black older adults without living kin in the United States, 2015 to 2060. Proc Natl Acad Sci.

[27] Uhlenberg P, Gierveld JD (2004). Age-segregation in later life: An examination of personal networks. Ageing Soc.

[28] Brummett BH, Barefoot JC, Siegler IC, Clapp-Channing NE, Lytle BL, Bosworth HB, Williams RB, Mark DB (2001). Characteristics of socially isolated patients with coronary artery disease who are at elevated risk for mortality. Psychosom Med.

[29] Cornwell EY, Waite LJ (2009). Social disconnectedness, perceived isolation, and health among older adults. J Health Soc Behav.

[30] Coyle CE, Dugan E (2012). Social isolation, loneliness and health among older adults. J Aging Health.

[31] Shankar A, McMunn A, Banks J, Steptoe A (2011). Loneliness, social isolation, and behavioral and biological health indicators in older adults. Health Psychol.

[32] Shaw BA, Krause N, Liang J, Bennett J (2007). Tracking changes in social relations throughout late life. J Gerontol B Psychol Sci Soc Sci.

[33] Uchino BN, Garvey TS (1997). The availability of social support reduces cardiovascular reactivity to acute psychological stress. J Behav Med.

[34] Penninx BW, Van Tilburg T, Kriegsman DM, Boeke AJ, Deeg DJ, Van Eijk JT (1999). Social network, social support, and loneliness in older persons with different chronic diseases. J Aging Health.

[35] Juvonen J, Schacter HL, Lessard LM (2021). Connecting electronically with friends to cope with isolation during COVID-19 pandemic. J Soc Pers Relat.

[36] Holman EA, Thompson RR, Garfin DR, Silver RC (2020). The unfolding COVID-19 pandemic: A probability-based, nationally representative study of mental health in the United States. Sci Adv.

[37] Ray CD (2021). The trajectory and determinants of loneliness during the early months of the COVID-19 pandemic in the United States. J Soc Pers Relat.

[38] Shaw P, Blizzard S, Shastri G, Kundzicz P, Curtis B, Ungar L, Koehly L (2021). A daily diary study into the effects on mental health of COVID-19 pandemic-related behaviors. Psychol Med.

[39] Lopez L, Hart LH, Katz MH (2021). Racial and ethnic health disparities related to COVID-19. JAMA.

[40] McCormack G, Avery C, Spitzer AK, Chandra A (2020). Economic vulnerability of households with essential workers. JAMA.

[41] Tai DB, Shah A, Doubeni CA, Sia IG, Wieland ML (2021). The disproportionate impact of COVID-19 on racial and ethnic minorities in the United States. Clin Infect Dis.

[42] Kliem S, Mößle T, Rehbein F, Hellmann DF, Zenger M, Brähler E (2015). A brief form of the Perceived Social Support Questionnaire (F-SozU) was developed, validated, and standardized. J Clin Epidemiol.

[43] Nikolaidis A, Paksarian D, Alexander L, Derosa J, Dunn J, Nielson DM, Droney I, Kang M, Douka I, Bromet E, Milham M (2021). The Coronavirus Health and Impact Survey (CRISIS) reveals reproducible correlates of pandemic-related mood states across the Atlantic. Sci Rep.

[44] Heckman JJ. Sample selection bias as a specification error. Econometrica. 1979;47(1):153–61.

[45] Lakey B, Cohen S, Cohen S, Underwood LG, Gottlieb BH (2000). Social support theory and measurement. Social support measurement and intervention: A guide for health and social scientists.

[46] Giebel C, Pulford D, Cooper C (2021). COVID-19-related social support service closures and mental well-being in older adults and those affected by dementia: a UK longitudinal survey. BMJ Open.

[47] Sommerlad A, Marston L, Huntley J, Livingston G, Lewis G, Steptoe A, Fancourt D (2021). Social relationships and depression during the COVID-19 lockdown: longitudinal analysis of the COVID-19 Social Study. Psychol Med.

[48] Stokes JP (1983). Predicting satisfaction with social support from social network structure. Am J Community Psychol.

[49] Silverio-Murillo A, Hoehn-Velasco L, Tirado AR, de la Miyar JR (2021). COVID-19 blues: Lockdowns and mental health-related google searches in Latin America. Soc Sci Med.

[50] Berkman LF, Glass T (2000). Social integration, social networks, social support, and health. Soc Epidemiol.

[51] Almquist YB, Landstedt E, Hammarström A (2017). Associations between social support and depressive symptoms: social causation or social selection—or both?. Eur J Public Health.

[52] Lang FR, Carstensen LL (1994). Close emotional relationships in late life: further support for proactive aging in the social domain. Psychol Aging.

